# A recent overview on sulforaphane as a dietary epigenetic modulator

**DOI:** 10.17179/excli2019-2039

**Published:** 2020-01-15

**Authors:** Tae Kyung Hyun

**Affiliations:** 1 Department of Industrial Plant Science and Technology, College of Agricultural, Life and Environmental Sciences, Chungbuk National University

## ⁯

***Dear Editor,***

Gene expression is mediated by chromatin epigenetic changes, including DNA methylation, histone modifications, promoter-enhancer interactions, and non-coding RNA (microRNA and long non-coding RNA)-mediated regulation (Chen et al., 2017[5]). Approximately 50 % of all tumor suppressor genes are inactivated through epigenetic modifications, rather than by genetic mechanisms, in sporadic cancers (Meeran et al., 2010[12]; Su et al., 2018[16]). Accumulating evidence suggests that epigenetic modulators are important tools to improve the efficacy of disease prevention strategies (Ratovitski, 2017[14]; Carlos-Reyes et al., 2019[4]; Hassan et al., 2019[8]). 

Sulforaphane ([1-isothioyanato-4-(methyl-sulfinyl)butane], SFN) is a naturally occurring, sulfur-containing isothiocyanate derivative that is found in the seeds and sprouts of cruciferous vegetables such as broccoli, cabbage, cauliflower, and kale (Vanduchova et al., 2019[19]). Because SFN induces the nuclear factor erythroid 2-related factor 2 (Nrf2)-antioxidant response element pathway that induces the cellular defense against oxidative stress (Trio et al., 2016[18]), SFN has received increased attention because it acts as an antioxidant, antimicrobial, anti-inflammatory, and anticancer agent (Vanduchova et al., 2019[19]). Various mechanisms, including apoptosis activation, nuclear factor-κB pathway inhibition, and cell cycle arrest induction, have been proposed to explain the beneficial effects of SFN in preventing multiple types of cancer (Tortorella et al., 2015[17]). Indeed, the increasing attention of SFN as an epigenetic modulator continues to contribute to new developments in clinical trials. 

This letter presents a summary of key recent studies investigating the function of SFN as an epigenetic modulator in several human diseases (Table 1(Tab. 1); References in Table 1: Abbas et al., 2016[1]; Ali Khan et al., 2015[2]; Cao et al., 2018[3]; Fisher et al., 2016[6]; Gao et al., 2018[7]; Lewinska et al., 2017[9]; Li et al., 2019[10]; Lubecka-Pietruszewska et al., 2015[11]; Okonkwo et al., 2018[13]; Royston et al., 2018[15]; Yang et al., 2015[20]; Yuan et al., 2018[21]; Zhao et al., 2018[22]; Zhou et al., 2019[23]). I believe that this letter will stimulate future research on the development of SFN as an epigenetic modulator for successful chemo-prevention and alternative therapeutic approaches. 

## Conflict of interest

The author declares no conflict of interest.

## Figures and Tables

**Table 1:**
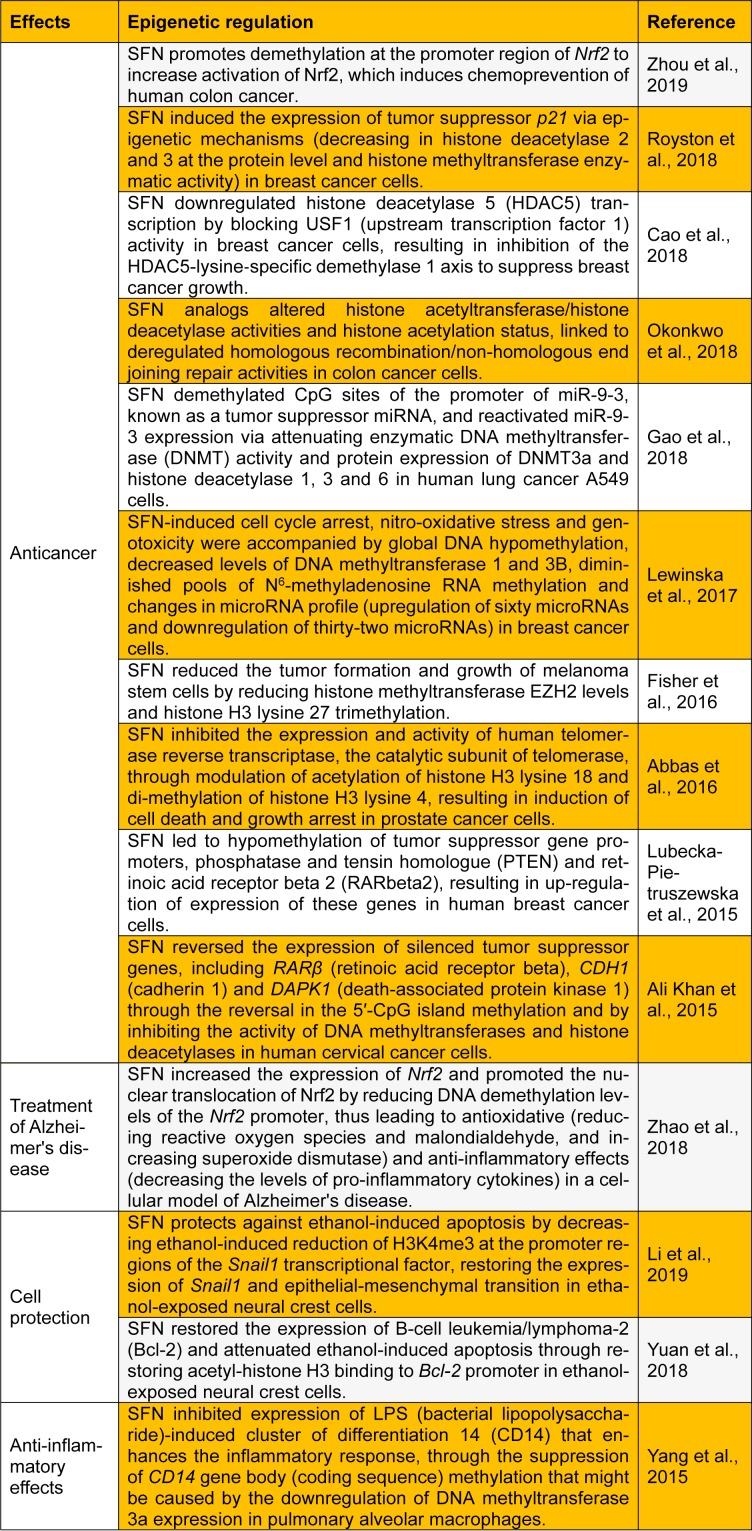
Recent updates on sulforaphane (SFN) as a dietary epigenetic modulator
